# ICPTC: Iranian commercial pistachio tree cultivars standard dataset

**DOI:** 10.1016/j.dib.2021.107348

**Published:** 2021-09-04

**Authors:** Ahmad Heidary-Sharifabad, Mohsen Sardari Zarchi, Gholamreza Zarei

**Affiliations:** a Department of Computer Engineering, Maybod Branch, Islamic Azad University, Maybod, Iran; b Department of Computer Engineering, Meybod University, Meybod, Iran; c Department of Agronomy, Maybod Branch, Islamic Azad University, Maybod, Iran

**Keywords:** Pistachio cultivars, Vernalization, Deep learning, Image classification, Plant classification

## Abstract

This paper contains datasets related to the “An efficient deep learning model for cultivar identification of a pistachio tree” [1]. There are about 11 species of pistachio that often have a high commercial and economic value in Iran and United States. The ability to identify pistachio tree cultivars, due to differences in the characteristics/traits of these species, is crucial for harvest the optimal yields, cost reduction, and damage prevention. For this purpose, identification of pistachio tree cultivars in their natural habitat is necessary. The cultivar identification relying on its appearance is a challenging vision task and can be facilitated by deep learning. The feasibility of applying deep learning algorithms to identify Pistachio tree cultivars depends on access to the appropriate relevant dataset.

Therefore, ICPTC dataset was collected from natural habitats of different trees of Pistachio cultivars, in real-world conditions from pistachio orchard farms of Chah-Afzal region in Ardakan County, Yazd, Iran. This imbalanced dataset is compiled of 526 RGB color images from 4 Pistachio tree cultivars, each cultivar 109-171 images. The tree of Iranian commercial pistachio cultivars, with names like Jumbo (Kalle-Ghuchi), Long (Ahmad-Aghaei), Round (O'hadi), and Super-long (Akbari) have distinctive branch expansion, leaf patterns, leaf shapes and colors. Imaging is performed from multiple trees for each cultivar, with different camera-to-target distances, viewpoints, angles, and natural sunlight during April and May in the spring. The collected images are not pre-processed, only grouped into their respective class (Jumbo, Long, Round, and Super long). The images in each class are separated by 20% for testing, 17% for validation, and 63% for training. Test images are selected from trees different from the training set. Then training and validation images are randomly separated from the remaining images in each category.

The ICPTC dataset is publicly and freely available at https://data.mendeley.com/datasets/6mmjjkpd5m/draft?a=af46a232-df30-4cf1-b303-6071d90ac8ad

## Specifications Table

**Table:** 

Subject	Computer Vision and Pattern Recognition, Deep Learning
Specific subject area	Image classification, Plant classification, *Pistachio* cultivar identification
Type of data	Images in RGB color space (JPG).
How data were acquired	Imaging is performed using Samsung SM-J701F cell phone camera with a 1: 1 (4.2-megapixel) 2160-by-2160 resolution.
Data format	Raw (unedited .JPG image files.) .jpeg
Parameters for data collection	Providing images of *Pistachio tree cultivars* under unconstrained conditions in their natural habitat.
Description of data collection	Imaging is performed on sunny days of spring at different times in real-world conditions. Images are collected in the natural habitat of *Pistachio tree cultivars* from various branches of multiple trees.
Data source location	*Pistachio* orchard farms of Chah-Afzal region in Ardakan County, Yazd, Iran. Study location geographical coordinates are 32° 29’ North, 53° 40’ East.
Data accessibility	Mendeley Data https://data.mendeley.com/datasets/6mmjjkpd5m/1 Code is available at https://doi.org/10.5281/zenodo.5172947
Related research article	Heidary-Sharifabad Ahmad, Sardari Zarchi Mohsen, Emadi Sima, Zarei Gholamreza, “An efficient deep learning model for cultivar identification of a pistachio tree”, British Food Journal, doi: 10.1108/BFJ-12-2020-1100

## Value of the Data

• The pistachio trees' cultivar identification based exclusively on their images is possible but is a challenging task even for specialists and orchardists. Image-based recognition of a Pistachio tree cultivar should be addressed by today's state-of-the-art deep learning approaches. This dataset is a resource for use by deep learning models and the computer vision community.
• This dataset can be used to advance fine-grained classification researches.
• Identification of cultivars by deep learning methods that can be done by using this data set is a breakthrough in horticulture.
• This dataset is small but contains high-resolution images, but to train a deep learning model, larger datasets and lower-resolution images are usually needed. To address this problem, several patches of training images can first be extracted and used to train a deep learning model. The main challenge here is applying an appropriate strategy to extract these patches. Instead, there is the opportunity to cost-effectively extend this dataset to other classes. Automatic Pistachio orchard analysis, including Pistachio tree cultivars identification in real-world, and imbalanced object classification using few high-resolution images might benefit from this dataset.
• The ICPTC is a complex multiclass image dataset for researchers in the deep learning community for the development of image classification using computer vision methods.
• This image dataset includes tree images under uncontrolled conditions with variations include rotation, viewpoint, illumination, and intra-cultivar.
• ICPTC dataset can serve as a motivation to encourage further research into computer vision methods for tree cultivar identification in the real world. Researchers can use it during the development of new deep algorithms.

## 1 Data Description

*Pistachio* is a deciduous, subtropical, and dioecious plant [2]. The genus of *pistachio* has 11 species, all of which secrete mastic or turpentine. The pistachio cultivars in the world are called domestic pistachios. These varieties produce nutrient nuts and other species are mostly used for turpentine production, ornamental, and a base for domestic pistachios [3]. The pistachio can be planted in different soil conditions and water due to its resistance to drought and salinity. The nut seed of *pistacia vera L*. is a popular edible because of its useful nutritional properties and pleasant taste. The pistachio among agricultural products as a strategic product has special economic importance in some countries, including Iran, the United States, and Turkey [4].

Each variety of pistachio cultivars has its characteristics and high-quality pistachio varieties are cultivated in Iran [2]. The main cultivations of pistachio in Iran are Jumbo (Kalleh-Ghuchi), Long (Ahmad-Aghaei), Round (O'hadi), and Super-long (Akbari) which are the major commercial varieties of pistachio in this country. The high yield and large size of the Jumbo cultivar made it popular. The Jumbo tree is with thick and strong branches. Jumbo cultivar is an early flowering variety and is susceptible to spring frost. This cultivar is vulnerable to nutrient and water deficiencies. The Round cultivar is resistant to pistachio psylla and compatible with most pistachio planting areas. The common pistachio psylla (*Agonoscena Pistaciae*) is one of the important and primary pests in pistachio orchards in Iran and the world. This insect is spread in all pistachio planting regions and causes significant damage to the pistachio crops annually. The amount of damage of this pest varies according to the pistachio tree cultivar [2]. The tree of this cultivar has a wide crown and an intermediate growth. The color of its leaf is darker than Jumbo, and the length of its terminal leaflet, and the length of its leaf, are longer than Jumbo. The terminal has a longer petiole and the leaflet is wide oval than the Jumbo cultivar. In most Iranian orchards, there are mixed these two cultivars, so it is very important to distinguish between Round and Jumbo cultivars. Among Iranian pistachio commercial cultivars, the super-long cultivar has the highest economic value. This cultivar has a heavy bearing in alternate years and high production potential and is susceptible to pest attacks and psylla. In this cultivar, the probability of damage caused by spring-frost is less than others because is late flowering, therefore tolerant to spring-frost. The Long cultivar has an extreme biennial bearing and high yield. Biennial bearing (Alternate bearing) is the irregular yielding of pistachio trees, from year to year. In the “high-yield” year or on-year, the production of pistachios is abundant and desirable, followed by a “low-yield” year or off-year. Some pistachio cultivars, such as the Long (Ahmad-Aghaei) cultivar, are genetically more biennial bearing and some cultivars, such as the Super-long (Akbari) cultivar, have less biennial bearing [2]. This cultivar's timely harvesting and its crop processing immediately are very important because is more susceptible to fungal growth than other aforementioned cultivars [2].

The term vernalization means the ability to flower in spring by being exposed to sufficient winter chilling. All pistachio tree cultivars need summer heat and winter chill [5]. Usually, the beginning of the dormancy period in pistachio trees is gradual, but leaving this period requires meeting their chilling needs. Meeting the chilling needs of pistachio trees leads to their homogeneous and symmetrical flowering. The chilling need (vernalization period) is determined by chilling hours that is the most common model [2]. But vernalization period or chilling requirement is not equal for all pistachio tree varieties [6]. In recent years, considering the warming globe, the lack of vernalization period is serious problems in pistachio growing areas, because ultimately will lead to very low yield [7]. A part of the chilling requirement can be compensated by applying some chemical substances. The amount and time of use of these substances are different for different cultivars of pistachios, so the correct identification of the tree cultivar is very important to prevent possible damage [1].

**Fig. 1:**
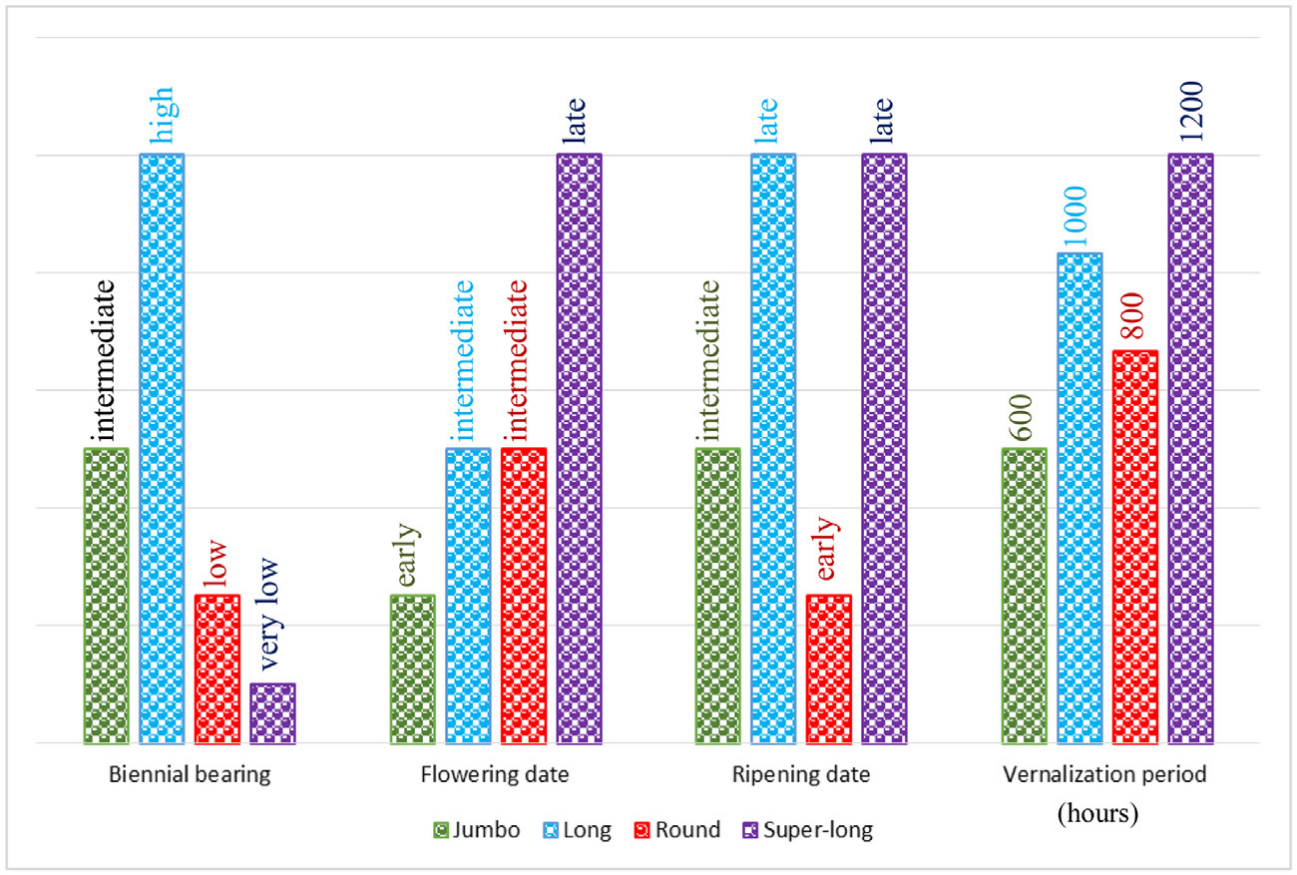
Different traits of Iranian commercial pistachio varieties.

Pistachio varieties are classified into early flowering, medium flowering, and late flowering classes depending on flowering time. Also, pistachio varieties are classified into very early ripening, early ripening, intermediate ripening, late-ripening, and very late ripening classes based on ripening time. During biennial bearing the crop fluctuates between an on-year of heavy crops followed by an off-year of no or little yields. Some of the traits of the studied cultivars are shown in Fig. 1 for comparison. Not knowing the pistachio cultivar, can cause irreparable damage. So, the pistachio trees cultivar recognition is crucial in the process of harvesting optimal yields as well as cost savings [1].

Deep learning can be used to automatic tree cultivar identification [8]. Applying deep learning techniques depends on the existence of a relevant dataset. Therefore, we collected the ICPTC (Iranian Commercial Pistachio Tree Cultivars) dataset containing 526 images of 4 different *Pistachio* tree cultivars. This image dataset was collected in real-world uncontrolled conditions using a usual imaging device during April and May in the spring.

**Fig. 2:**
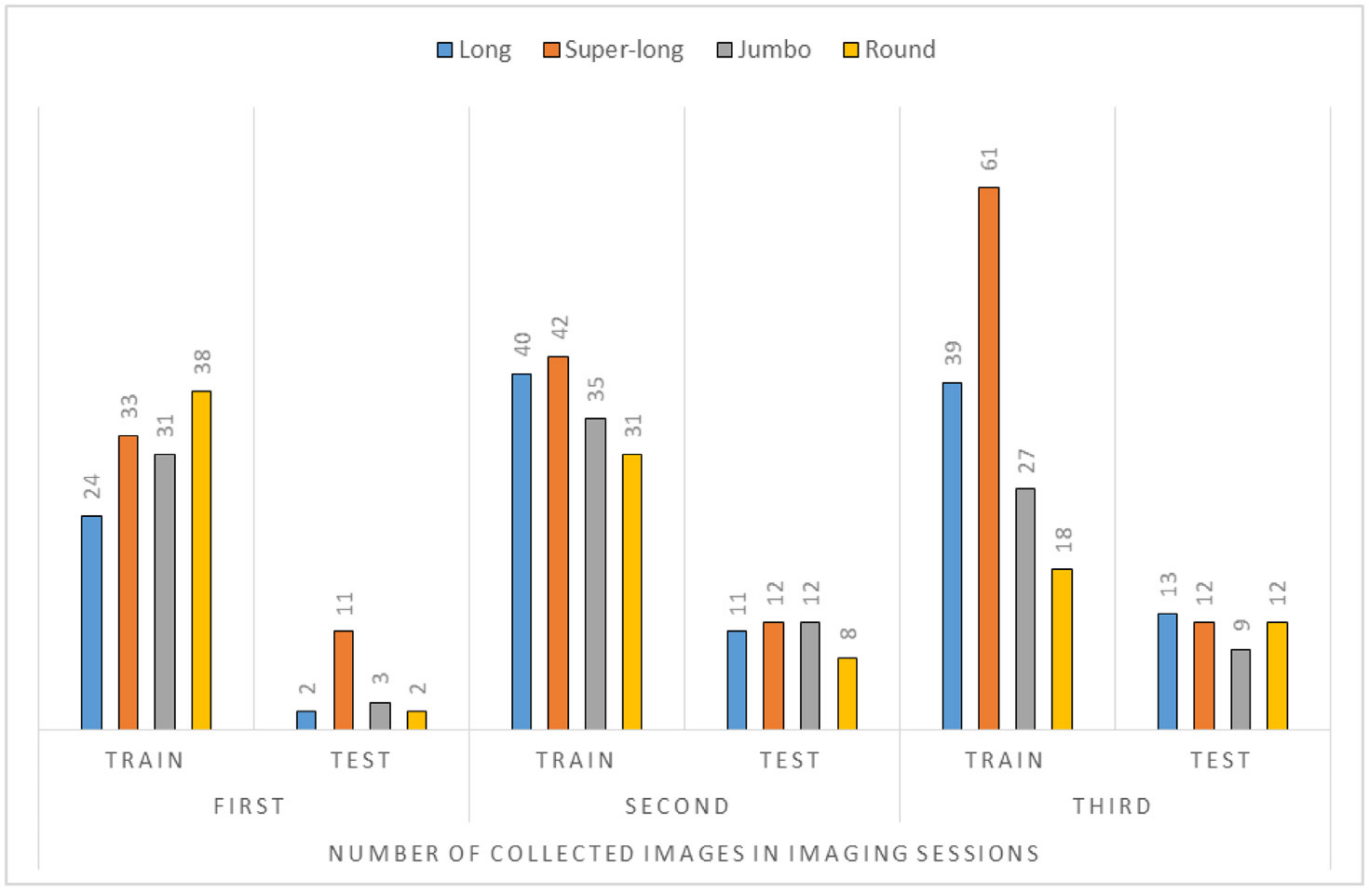
The ICPTC dataset collecting details.

To create a class name the first letters of the cultivar name were used. The different numbers of images that were collected for each cultivar were also listed in this table. First, we put all the collected images in the photo sessions in a separate folder for each class, then we randomly identified 20% of the images in each folder as test images. 20% of collected images for each class were separated into test set that is often from distinct trees from others, 63% randomly were separated to the training set, and the remaining 17% were assigned to the validation set. The images collected were not pre-processed, only they were placed in the appropriate folders and classes. In three imaging sessions (early April, middle April, and middle May), the ICPTC dataset was collected. These images were collected at the time which there was enough natural light, in the middle of the day. Fig. 2 shows some details on ICPTC collecting.

**Fig. 3:**
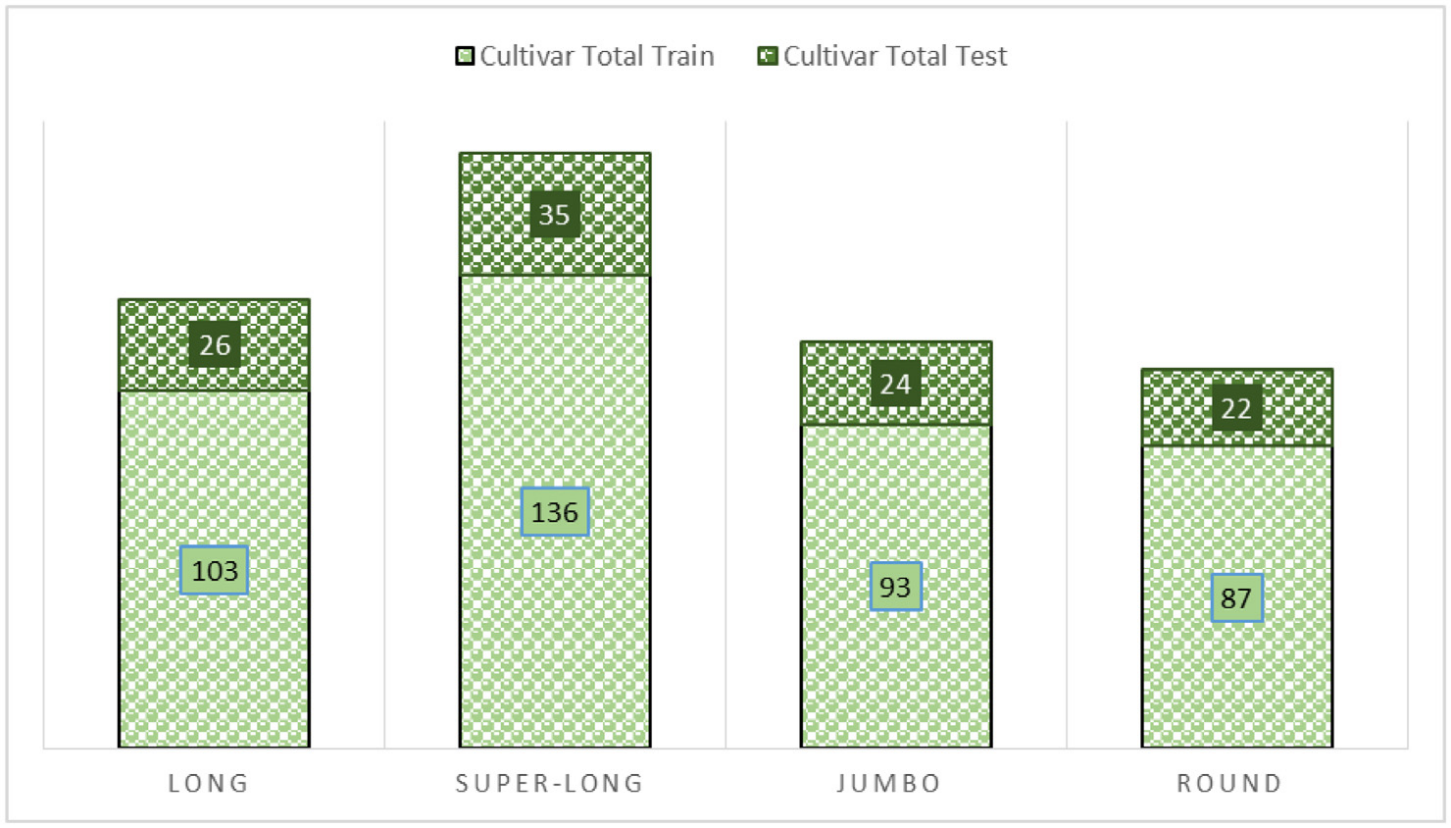
The ICPTC dataset total images.

The total images that collected for each cultivar are shown in Fig. 3.

**Fig. 4:**
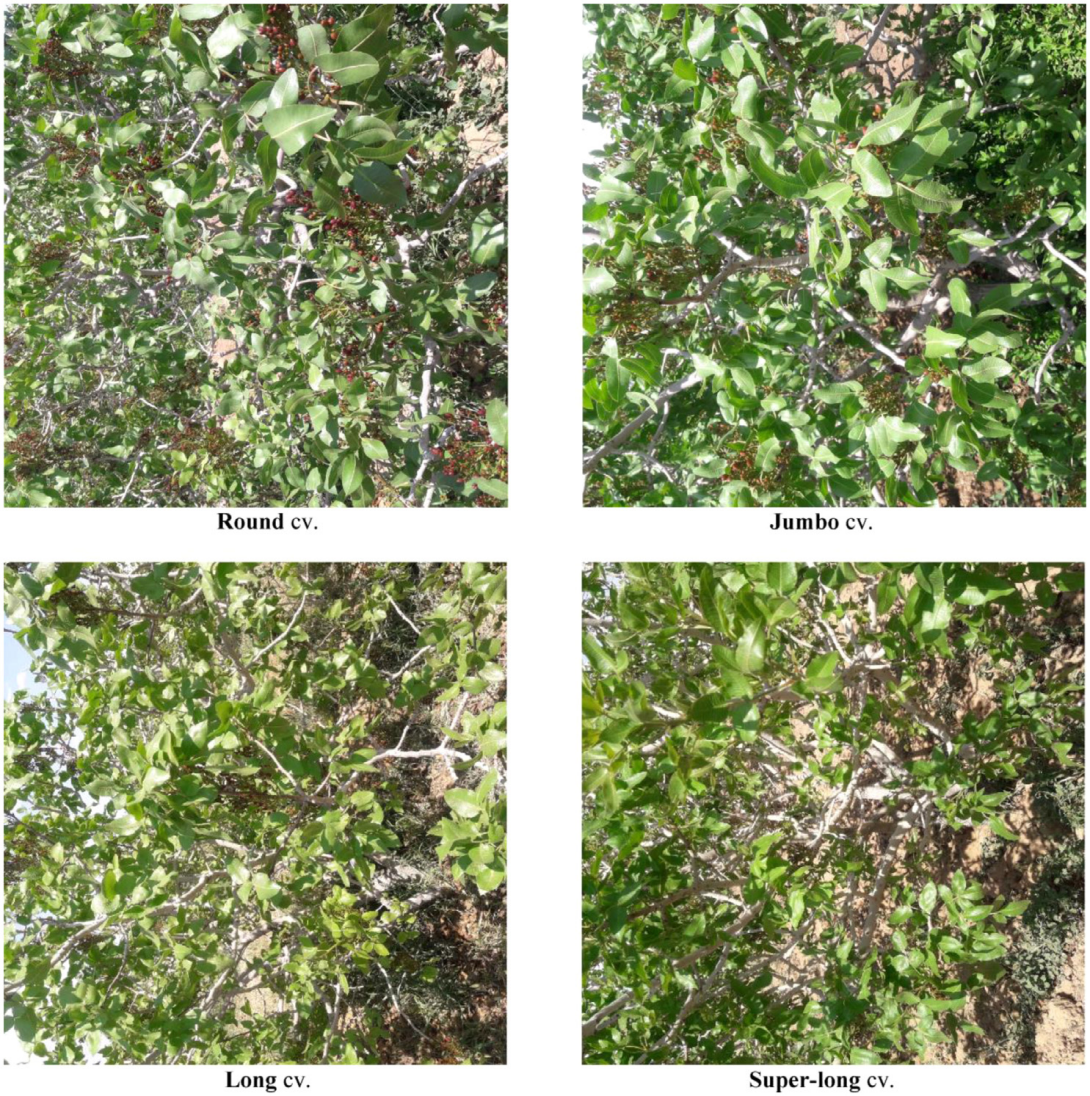
One sample image from each Pistachio tree cultivar included in the ICPTC dataset.

One sample image from each *ICPTC dataset* class was shown in Fig. 4.

## 2 Experimental Design, Materials and Methods

Initially, four orchards in the studied area each with one of the studied pistachio cultivars were chosen. The age of the trees in the chosen orchards was almost the same.

1. Camera setting and specification

Imaging is performed using a cell phone camera:

The Samsung SM-J701F cell phone camera with a 1: 1 (4.2-megapixel) 2160-by-2160 resolution.

The camera was utilized for image collection in natural light during sunny days.

2. Imaging time and conditions

Studied *Pistachio* cultivars often have leaves and flowers in the spring, hence imaging is performed in April and May in their habitat. Outdoors and nature have many uncontrollable factors affecting images, such as light intensity throughout the day, therefore Imaging was performed at times of sunny days in natural sunlight. Some other factors also affect acquired images, such as viewing angles, and camera-to-target distances. The following conditions are considered to collect images:

• From each intended tree only one image is taken.
• The background of the taken image should include the foliage of the intended tree, as far as possible.
• We consider the desired quality of the acquired images. In capturing an image there is enough light, and the shooting angle is appropriate.
• We took the image from a suitable distance from the intended tree to the foliage of the adjacent trees that are not included in the image, and more foliage of the intended tree is included.

3. ICPTC dataset in a repository

The ICPTC dataset is available online at Mendeley Repertory. It is structured in one main folder (ICPTC_size2160), the main folder contains all cultivars images in three zipped files: test.zip contains test images, train.zip contains training images, and validation.zip contains validation images. In each of these zipped files, 4 subfolders were named class names, each contains images in that class. The ICPTC specification table and figure of sample images are also included in the main folder.

4. ICPTC dataset classification

We investigate the efficiency of the ICPTC dataset by a deep learning model. In our proposed patch-based model, the ORB image features are first detected and the image patches that containing these features are extracted. These patches are used to train EfficientNet-B1 [9] deep learning model. Then, to predict an image, image patches containing ORB are extracted and predicted using the aforementioned trained deep model. The prediction scores of image patches are sorted by high score, and then their average is calculated. To enhance prediction accuracy grid-based patches are also extracted and used. To see more details on the proposed model please refer to the related research paper [1].

## Supplementary Material

The ICPTC dataset can be found in the online version at https://data.mendeley.com/datasets/6mmjjkpd5m/1

## Ethical Considerations

Researchers are mindful of the fact that no confidential data or private sources have been used and no harm has been done to the chosen pistachio trees or orchards.

## CRediT authorship contribution statement

**Ahmad Heidary-Sharifabad:** Conceptualization, Methodology, Software, Investigation, Writing – review & editing. **Mohsen Sardari Zarchi:** Supervision, Validation, Writing – original draft. **Gholamreza Zarei:** Visualization, Data curation, Resources.

## Declaration of Competing Interest

The authors declare that they have no known competing financial interests or personal relationships which have, or could be perceived to have, influenced the work reported in this article.
